# A Chitosan-Based Micellar System as Nanocarrier For the Delivery of Paclitaxel

**DOI:** 10.3390/polym12020380

**Published:** 2020-02-08

**Authors:** Yang Han, Na Liang, Pengfei Yan, Yoshiaki Kawashima, Fude Cui, Shaoping Sun

**Affiliations:** 1 Department of Pharmaceutical Engineering, School of Chemistry and Material Science, Heilongjiang University, Harbin 150080, China; hanyang000626@163.com (Y.H.); yanpf@vip.sina.com (P.Y.); 2 College of Chemistry & Chemical Engineering, Harbin Normal University, Harbin 150025, China; 3 Department of Pharmaceutical Engineering, School of Pharmacy, Aichi Gakuin University, Nagoya 464-8650, Japan; sykawa123@163.com; 4 School of Pharmacy, Shenyang Pharmaceutical University, Shenyang 110016, China; syphucuifude@163.com

**Keywords:** chitosan, cholesterol, micelles, mPEG, redox-responsive

## Abstract

In this study, a redox-sensitive chitosan derivative with modifications by cholesterol, sulfhydryl, and mPEG (mPEG-CS(SH)-CHO) was successfully synthesized and characterized. Due to its amphiphilicity, the conjugate could spontaneously form micelles in an aqueous environment. The optimized paclitaxel (PTX)-loaded mPEG-CS(SH)-CHO micelles, with a mean diameter of 158 nm, zeta potential of +26.9 mV, drug loading of 11.7%, and entrapment efficiency of 88.3%, were successfully prepared. The results of an XRD study demonstrated that PTX was loaded in the core of the micelles in a non-crystalline state. Inspiringly, the PTX-loaded micelles possessed excellent anticancer effect but low toxicity to the body. It can be concluded that the mPEG-CS(SH)-CHO micellar system is a promising drug delivery carrier for the controlled release of PTX.

## 1. Introduction

Cancer is one of the most devastating malignant diseases in the world. As an effective chemotherapeutic drug, paclitaxel (PTX) has been widely applied to treat various kinds of cancers, such as breast cancer, ovarian cancer, and non-small-cell lung cancer [1]. However, due to its low solubility in aqueous solution and the lack of selectivity to cancer cells, the clinical use of PTX is limited, and even associated with severe side effects like cardiotoxicity, nephrotoxicity, and hypersensitivity [2]. The development of a superior PTX delivery system with excellent antitumor activity and low toxicity to normal tissues is urgently needed.

Recently, stimuli-responsive micelles have gained a widespread interest for their outstanding advantages [3]. More specifically, the micelles formed from amphiphilic copolymers can effectively encapsulate the hydrophobic drug in the hydrophobic inner core, and the hydrophilic outer shell can improve the compatibility of the system [4]. Due to the leaky vasculature in solid tumor tissues, the nanosized micelles can efficiently accumulate within tumor sites via the enhanced permeability and retention (EPR) effect [5]. Moreover, it is well known that there are large variations in physiological conditions between normal tissues and the tumor sites, such as temperature, pH, redox property, or enzyme levels [6]. The endogenous stimuli can be utilized to trigger the drug release within the diseased area, and this would increase the concentration of drugs in the tumor, whereas normal tissues are left unaffected [7].

Among various stimuli, redox potential has been highly studied because of the significant difference in glutathione (GSH) concentration between the mildly oxidizing extracellular milieu (approximately 2–10 μM in the plasma) and the reducing tumor intracellular microenvironment (approximately 2–10 mM in the cytoplasm) [8]. Redox-sensitive polymers usually contain disulfide bonds in the molecules. The disulfide bonds-employing nanoparticles are stable at normal physiologic conditions and tend to effectively release the drugs within tumor cells [9]. The tumor-specific drug release enables the redox-sensitive nanoparticles to display enhanced antitumor efficacy and reduced toxicity. *N*-acetyl-*L*-cysteine (NAC), as a cysteine derivative, has free thiol groups (-SH) in the molecules, and there will be stable disulfide bonds formed between two NAC molecules [10]. Therefore, the introduction of NAC could endow the polymer with redox-sensitivity.

Furthermore, to be an ideal carrier, the polymeric micellar system should avoid the uptake by the reticuloendothelial system (RES) and possess a long circulation time to increase the passive accumulation in tumor tissues [11]. The PEGylation is a good choice to overcome these issues [12]. As one of the most common hydrophilic modifiers, methoxy poly (ethylene glycol) (mPEG) is widely used for fabricating biocompatible nanoparticles. It is noteworthy that polymeric micelles modified by mPEG possess increased stability, prolonged half-life, and reduced uptake by the RES [13]. 

To date, many kinds of amphiphilic copolymers have been developed as micellar drug delivery carriers [14]. Chitosan (CS), as a natural polysaccharide, has been widely applied for its biocompatibility, biodegradability, and non-toxicity [15]. The CS with a high degree of deacetylation and low molecular weight is soluble in aqueous milieu, and it can be chemically modified rather easily, due to the hydroxyl groups and primary amine groups in the molecules [16]. In addition, chitosan-based nanocarriers can increase the paracellular permeability and thus enhance drug absorption [17]. Based on the above information, CS is a superior hydrophilic segment for amphiphilic copolymer synthesis.

Cholesterol (CHO) is an essential structural component that forms cell membranes [18]. As an endogenous substance synthesized by the liver, CHO has no cytotoxicity or immunogenicity [19]. It is usually used to modify materials due to its hydrophobicity and rigidity [20]. Furthermore, malignant cells consume large amounts of CHO and overexpress the low-density lipoprotein (LDL) receptors (which mediate the cholesterol transport pathway) due to their rapid proliferation [21]. Recent studies have demonstrated that CHO conjugates can be used as tumor-targeting carriers [22].

Recently, many chitosan derivatives have been developed for anti-cancer drug delivery. For instance, Jang et al. prepared core-shell type nanoparticles using cholesterol and PEG conjugated chitosan, and the drug loaded in the nanoparticles was released sustainedly [23]. Qu et al. synthesized a series of PEG modified N-octyl-O-sulfate chitosan to assemble micelles, and the micelles had increased circulation time [24]. In this work, chitosan modified by mPEG, CHO, and NAC (mPEG-CS(SH)-CHO) was synthesized for the first time and used for PTX delivery. It was speculated that the amphiphilic polymer could spontaneously form micelles in aqueous milieu, and PTX could be encapsulated into the hydrophobic core for intravenous injection. Due to the mPEG corona, the micelles could escape rapid clearance by the RES and enhance the accumulation in the tumor. The thiolation by NAC could produce a redox-responsive drug carrier. The micellar system could release the drug rapidly in the reducing environment of the tumor while remaining stable during the circulation of blood. This would improve the antitumor effect of the drug and reduce the toxicity to the body. The preparation of the polymer, the fabrication of the PTX-loaded mPEG-CS(SH)-CHO micelles, and a series of evaluations of the drug delivery system were elaborated in this paper.

## 2. Materials and Methods

### 2.1. Materials

Chitosan (M_w_ = 30 kDa, degree of deacetylation > 97%) was obtained from Kittolife Co., Ltd., Seoul, Korea. Cholesterol (CHO), succinic anhydride (SA), methoxy poly (ethylene glycol) (mPEG, M_w_ = 1900 Da), *N*-acetyl-*L*-cysteine (NAC), 1-(3-dimethylaminopropyl)-3-ethylcarbodiimide hydrochloride (EDC·HCl), N-hydroxysuccinimide (NHS), and Cremophor EL were obtained from Aladdin Industrial Co., Shanghai, China. Pyrene, 2,4,6-trinitrobenzene sulfonic acid (TNBS), and 3-(4,5-dimethylthiazol-2-yl)-2,5-diphenyl tetrazolium bromide (MTT) were provided by Sigma Chemical Co., St. Louis, MO, USA. Paclitaxel (PTX, purity of 99.9%) was obtained from Natural Field Biological Technology Co., Ltd., Xi’an, China. Dulbecco’s modified Eagle’s medium (DMEM), fetal bovine serum (FBS), bovine serum albumin (BSA), and phosphate buffered saline (PBS) were purchased from Gibco BRL, Carlsbad, CA, USA. All other solvents and chemicals were used without further purification.

### 2.2. Synthesis of mPEG-CS(SH)-CHO

The mPEG-CS(SH)-CHO was synthesized by a four-step reaction. First, cholesterol was modified by succinic anhydride (SA) to form the mono cholesteryl succinate (CHO-COOH). Next, mPEG was grafted onto chitosan to get mPEG modified chitosan (mPEG-CS). Then, the CHO-COOH was combined with mPEG-CS to form cholesterol modified mPEG-CS (mPEG-CS-CHO). Finally, the mPEG-CS(SH)-CHO was prepared by the reaction between NAC and mPEG-CS-CHO.

#### 2.2.1. Synthesis of CHO-COOH

Mono cholesteryl succinate was prepared according to the reported method [25]. Briefly, 200 mg of CHO and 200 mg of SA were dissolved in 2 mL of pyridine. The mixture was stirred at 45 °C for 72 h and then poured into the iced dilute hydrochloric acid solution. The CHO-COOH was precipitated and purified by recrystallization in the mixture of ethyl acetate and ethanol (*v*/*v* = 1:1). After oven-drying at 25 °C, the CHO-COOH powder was obtained. 

#### 2.2.2. Synthesis of mPEG-CS

The mPEG-CS was prepared by coupling mPEG with CS. In detail, 120 mg of CS was added to the mixture of distilled water (5 mL) and acetic acid (4 mL). Afterwards, 10 mL of mPEG solution (0.48 mg/mL) and 20 mL of formaldehyde (37%, m/v) were added. The mixture was stirred for 1 h at ambient temperature and then dialyzed against distilled water with molecular weight cut-off (MWCO) of 30 kDa for 48 h to remove the impurities. The product mPEG-CS was obtained by lyophilization. The yield of mPEG-CS was 81.3%.

#### 2.2.3. Synthesis of mPEG-CS-CHO

The mPEG-CS-CHO was synthesized by conjugating CHO-COOH with mPEG-CS. Briefly, 27 mg of CHO-COOH, 16 mg of EDC and 10 mg of NHS were dissolved in 1 mL of DMF. The solution was stirred for 2 h at 45 °C. Afterwards, the mPEG-CS solution with 150 mg of mPEG-CS dissolved in DMF was added to the above mixture drop by drop under mechanical agitation, and the mixture was further stirred for 72 h at 45 °C. The resultant was purified by dialysis against water for 24 h (MWCO of 30 kDa). The dialyzed solution was freeze-dried to get the mPEG-CS-CHO powder. The yield of this step was 83.7%.

#### 2.2.4. Synthesis of mPEG-CS(SH)-CHO

The thiolation of mPEG-CS-CHO was conducted by conjugating NAC to mPEG-CS-CHO. In brief, 10 mg of NAC was dissolved in 10 mL of DMF, and then 11 mg of NHS and 16 mg of EDC were added to activate carboxyl groups of NAC. After 2 h, 180 mg of mPEG-CS-CHO was added, and the mixture was stirred for 72 h at 45 °C. After reaction, the resultant was dialyzed against distilled water (MWCO of 30 kDa) for 36 h to remove the water-soluble impurities and the unreacted reagent. The product mPEG-CS(SH)-CHO was obtained from lyophilization. To protect the sulfhydryl groups from being oxidized, all processes were conducted under N_2_ atmosphere. The yield of this reaction step was 86.5%.

### 2.3. Characterization of mPEG-CS(SH)-CHO

Fourier-transform infrared (FT-IR) spectra of CS, mPEG-CS, mPEG-CS-CHO, and mPEG-CS(SH)-CHO were recorded by an FT-IR Tensor II spectrometer (Bruker, Zurich, Switzerland) to confirm the formation of mPEG-CS(SH)-CHO. Moreover, the proton nuclear magnetic resonance (^1^H NMR) study was also performed on a Bruker AV-400 spectrometer (Bruker, Zurich, Switzerland) using DMSO-d6 as the solvent. The critical micelle concentration (CMC) of mPEG-CS(SH)-CHO and the substitution degree of mPEG, CHO, and NAC were determined according to the previous report [26].

### 2.4. Preparation and Characterization of the mPEG-CS(SH)-CHO Micelles

The PTX-loaded mPEG-CS(SH)-CHO micelles were fabricated by using an ultrasonic method. Typically, 1.5 mL of the PTX in acetone solution (0.25 mg/mL) was added to 10 mL of the mPEG-CS(SH)-CHO solution (0.25 mg/mL in distilled water) under ultrasonication at 300 W for 3 min in an ice bath (pulse on for 3 s and off for 2 s). Afterwards, the mixture was dialyzed against water for 2 h (MWCO of 30 kDa), and then the unloaded PTX was removed by centrifugation at 4000 rpm for 10 min. The resultant supernatant was freeze-dried to obtain the mPEG-CS(SH)-CHO micelles. The bare micelles were prepared without PTX addition. 

The drug loading and entrapment efficiency of the mPEG-CS(SH)-CHO micelles were investigated by using the HPLC method (reverse-phase column: Diamonsil^TM^, 4.6 × 200 mm, 5 μm; mobile phase delivery pump: LC-16, Shimadzu, Tokyo, Japan; mobile phase: acetonitrile/H_2_O = 70/30, *v*/*v*; detection wavelength: 227 nm; column temperature: 35 °C).

The zeta potential and hydrodynamic diameter of the PTX-loaded mPEG-CS(SH)-CHO micelles were measured using the dynamic light scattering (DLS) method (Malvern Zetasizer Nano-ZS90 Instruments, Malvern, UK). A transmission electron microscope (TEM, H-7650, Hitachi Ltd., Tokyo, Japan) was applied to observe and image the morphology of the PTX-loaded micelles.

An X-ray diffraction (XRD) study was performed to investigate the crystalline characteristics change of PTX after incorporation into the micelles (Geigerflex, Rigaku Co., Tokyo, Japan). 

### 2.5. Protein Adsorption Tests

To evaluate the effect of mPEG on the stability of mPEG-CS(SH)-CHO micelles, protein adsorption tests were conducted as follows. A series of bovine serum albumin (BSA) in PBS 7.4 solutions were prepared, with the concentration ranging from 0.1 to 0.7 mg/mL. The fluorescence spectra of the BSA solution were recorded on a fluorescence spectrometer (RF-6000, Shimadzu, Tokyo, Japan) with an excitation wavelength of 285 nm. To obtain the standard curve of BSA, the absorbance value of each sample measured at 337 nm was plotted against BSA concentration. Afterwards, the mPEG, CS, CS(SH)-CHO, and mPEG-CS(SH)-CHO solutions were prepared, respectively, and the BSA concentration was set at 0.5 mg/mL. Samples were kept in an incubator at 37 °C for 3 h. The resulting protein-micelle complex was centrifuged at 18,000 rpm for 15 min. The resultant supernatant was analyzed, and the protein absorbance percentage of each sample was calculated.

### 2.6. In Vitro Drug Release Kinetics

The in vitro drug release study was conducted under different GSH concentrations. One milliliter of the micelles was put into a dialysis tube (MWCO of 30 kDa), and the tube was immersed in 20 mL of PBS 7.4 (with 10 mM GSH or 10 μM GSH). To provide sink conditions for PTX, 0.5% (*w*/*v*) Tween 80 was added to the release medium. The vessel was gently shaken in a shaker (SHA-B, Guohua Instrument, Changzhou, China) at 100 rpm and 37 ± 0.5 °C. At the scheduled time point, the medium was withdrawn and replaced with a fresh medium. The released PTX was assayed by the above HPLC method. 

### 2.7. In Vitro Cytotoxicity

The cytotoxicity of PTX-loaded mPEG-CS(SH)-CHO micelles against MCF-7 cells was assessed in vitro by MTT assay in comparison with PBMC cells (peripheral blood mononuclear cells, normal cells). Cells were cultivated in 96-well culture plates at the density of 1 × 10^4^ cells per well and incubated in 200 μL of DMEM media containing 10% FBS for 24 h (5% CO_2_, 37 °C). Then, the cells were incubated with different levels of PTX-loaded mPEG-CS(SH)-CHO micelles, the commercial formulation Taxol, and the blank micelles. After incubation for 24 h, 10 μL of MTT solution was added. Four hours later, the unreacted MTT was aspirated away, and 100 μL of DMSO was added to each well to dissolve the resulting formazan crystals. The absorbance at 490 nm was measured using a BioRad microplate reader (Bio-Rad 680, Bio-Rad Laboratories, Hercules, CA, USA). The cell viability was evaluated using the following formula:

*Cell viability* (%) = *A_sample_*/*A_control_* × 100% (1)

where *A_sample_* was the absorbance of the sample and *A_control_* was the absorbance of the control.

### 2.8. In Vivo Antitumor Efficacy

Kunming mice weighing 20 ± 2 g were obtained from Harbin Medical University, China, and housed in pathogen-free conditions with free access to murine chow and water (22 ± 2 °C, R.H. 50 ± 5%). All animal studies complied with the protocol approved by the Animal Ethics Committee of Heilongjiang University (190408001, approval date: April 8, 2019). 

To establish a tumor-bearing mice model, H22 cells (2 × 10^6^ cells) were injected subcutaneously into the right armpit of each mouse. When tumors became palpable (set as day 0), the mice were randomly divided into 3 groups (n = 6). Normal saline (model group), Taxol formulation (positive control, PTX 15 mg/kg), and the PTX-loaded mPEG-CS(SH)-CHO micelles (PTX 15 mg/kg) were administered intravenously on days 0, 3, 6, and 9. The tumor size was measured, and the tumor volume was estimated as follows:

*Volume* = 1/2 × *Length* × *Width*^2^ (2)

where *Length* and *Width* were the longest diameter and the shortest diameter of the tumor, respectively.

After the treatment, on day 12, all the animals were sacrificed. The tumor tissues were removed and weighed. The antitumor efficacy was evaluated using the index of tumor inhibition rate (TIR), which was calculated as follows:

*TIR* (%) = (*W_model_* − *W_treated_*)/*W_model_* × 100% (3)

where *W_model_* represented the average tumor weight of the model group and *W_treated_* represented the average tumor weight of the treated group.

### 2.9. Statistical Analysis

All experiments were performed in triplicate. Data were expressed as the mean ± standard deviation (SD). A student’s *t*-test was performed to assess the statistical significance, and *p* < 0.05 was considered statistically significant.

## 3. Results and Discussion

### 3.1. Preparation of mPEG-CS(SH)-CHO

**Figure 1:**
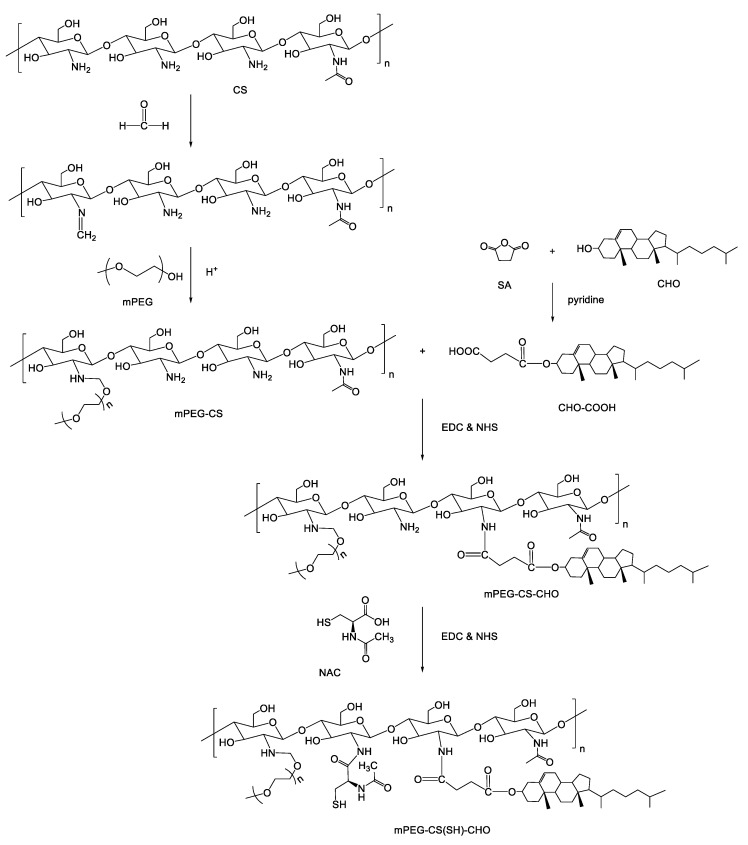
Synthetic route of mPEG-CS(SH)-CHO.

In this study, the chemical conjugate of mPEG-CS(SH)-CHO was synthesized via a series of coupling reactions. The synthetic scheme is shown in Figure 1. First, mono cholesteryl succinate (CHO-COOH) was prepared by coupling CHO with SA in the presence of pyridine, which was used not only as the solvent but also as the catalyst. Next, mPEG-CS was synthesized in a slightly acidic environment, where the primary amino groups of CS could be protonated and react with formaldehyde to form an intermediate containing carbon-nitrogen double bonds (C=N). Then, mPEG-CS was formed via the ether bonds between the intermediate and the hydroxyl groups of mPEG. Afterwards, mPEG-CS-CHO was prepared by linking mono cholesteryl succinate to mPEG-CS through the formation of amide bonds. The introduction of NAC to mPEG-CS-CHO was also based on the formation of amide bonds, and mPEG-CS(SH)-CHO was finally formed. The substitution degree of mPEG, CHO, and NAC were calculated as 13.6%, 8.7%, and 12.2%, respectively. 

### 3.2. Characterization of mPEG-CS(SH)-CHO

#### 3.2.1. FT-IR Characterization

**Figure 2:**
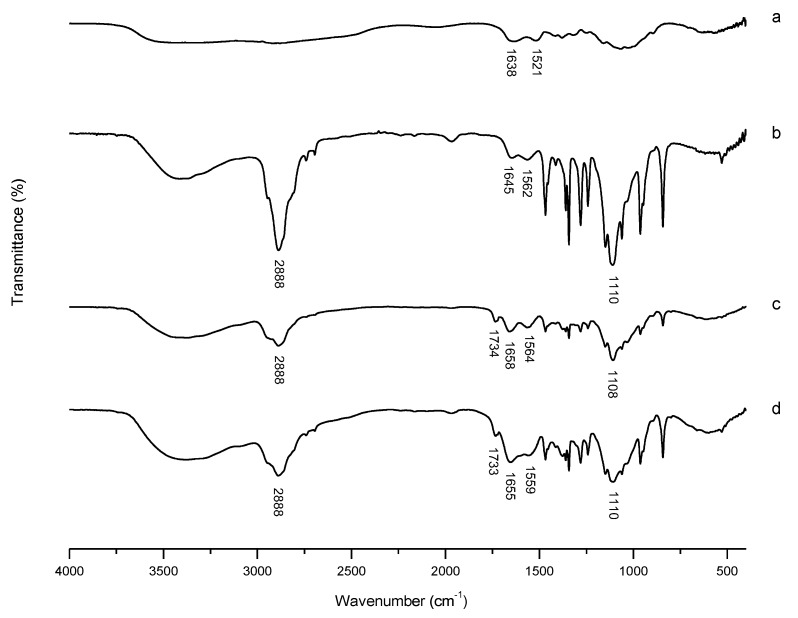
FT-IR spectra of (**a**) chitosan (CS), (**b**) mPEG-CS, (**c**) mPEG-CS-CHO, and (**d**) mPEG-CS(SH)-CHO.

FT-IR spectra were used to prove the successful formation of mPEG-CS(SH)-CHO. As presented in Figure 2, for CS, peaks at 1638 cm^−1^ and 1521 cm^−1^ were assigned to the stretching vibration of C=O (amide I band) and bending vibration of N-H (amide II band), respectively [27]. For mPEG-CS, new peaks at 2888 cm^−1^ and 1110 cm^−1^ were attributed to the stretching vibrations of –CH_2_– and C-O-C of mPEG, respectively, which indicated the introduction of mPEG [28]. As for mPEG-CS-CHO, the related changes in amide peaks (from 1645 and 1562 cm^−1^ to 1658 and 1564 cm^−1^, respectively) demonstrated the successful formation of new amido links. Moreover, the characteristic peak corresponding to the stretching vibration of C=O at 1734 cm^−1^ demonstrated the successful introduction of mono cholesteryl succinate. For mPEG-CS(SH)-CHO, the amide peaks shifted to 1655 cm^−1^ and 1555 cm^−1^, respectively. The shifts could be explained by the modification with NAC via the formation of new amide linkage. The differences mentioned above demonstrated the successful synthesis of mPEG-CS(SH)-CHO.

#### 3.2.2. ^1^H-NMR Characterization

**Figure 3:**
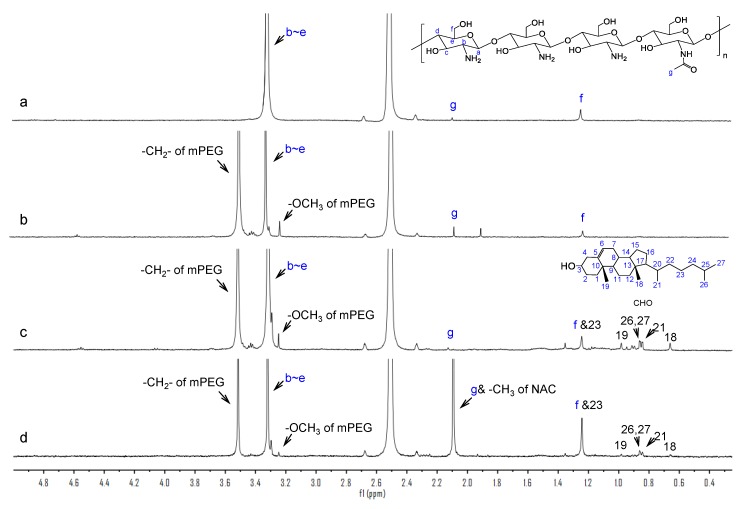
^1^H-NMR spectra of (**a**) CS, (**b**) mPEG-CS, (**c**) mPEG-CS-CHO, and (**d**) mPEG-CS(SH)-CHO.

The conjugate formation was further confirmed by ^1^H-NMR spectra. As illustrated in Figure 3, in the spectrum of CS, signals at 3.32 ppm, 2.10 ppm, and 1.25 ppm were ascribed to the H (b-e) on the glucosamine ring, three protons of N-acetyl glucosamine [29], and the hydroxymethyl groups [30], respectively. For mPEG-CS, the peaks appearing at 3.25 ppm and 3.51 ppm corresponded to the -OCH_3_ and methylene protons of mPEG, respectively. Compared with mPEG-CS, the typical signals in the spectrum of mPEG-CS-CHO at 0.66 ppm, 0.84 ppm, 0.86 ppm, and 0.98 ppm were due to the angular methyl protons of CHO (carbons 18, 21, 26 and 27, and 19) [31]. In addition, the enhanced signal at 1.25 ppm was attributed to the methylene protons of CHO (carbons 23). For mPEG-CS(SH)-CHO, the enhanced methyl peak at 2.10 ppm demonstrated the introduction of NAC, for there were also –CH_3_ in the molecules of NAC. The above results further certified the successful synthesis of mPEG-CS(SH)-CHO.

### 3.3. Preparation of mPEG-CS(SH)-CHO Micelles

As an amphiphilic copolymer, mPEG-CS(SH)-CHO could self-assemble into nanosized micelles in aqueous media. The hydrophobic part of CHO could serve as the internal core under the attractive force, and PTX could be encapsulated in the hydrophobic domains via the hydrophobic interactions or Van der Waals forces [32]. The hydrophilic mPEG and CS segments could be used as the outer shell. Moreover, the sulfhydryl groups of mPEG-CS(SH)-CHO formed disulfide bonds during the preparation of micelles. The disulfide bonds make the structure of micelles more stable, which might prevent the drug from being released prematurely into the blood stream. The CMC of mPEG-CS(SH)-CHO was determined to be 7.26 × 10^−3^ mg/mL, which indicated the high stability of mPEG-CS(SH)-CHO micelles at dilute conditions. For the optimized PTX-loaded mPEG-CS(SH)-CHO micelles, the drug loading capacity reached 11.7%, and the drug encapsulation efficiency was as high as 88.3%.

### 3.4. Characterization of PTX-Loaded mPEG-CS(SH)-CHO Micelles

#### 3.4.1. Hydrodynamic Diameter and Zeta Potential

**Figure 4:**
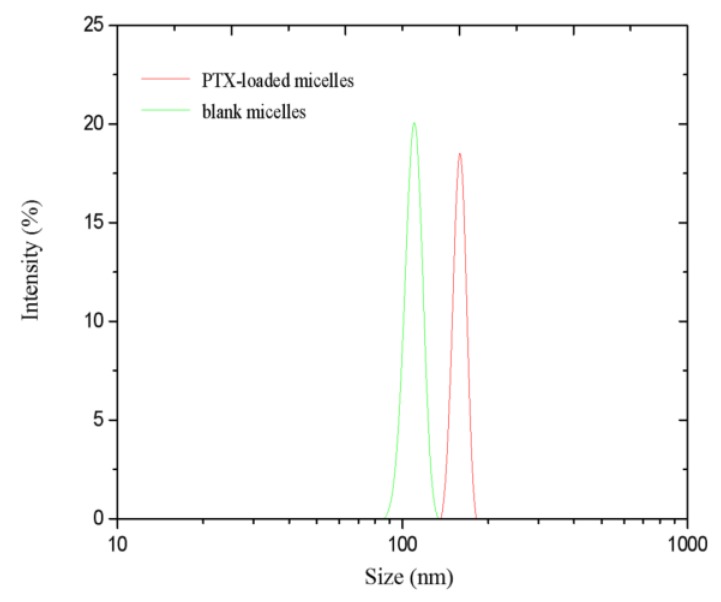
Size distributions of the blank micelles and paclitaxel (PTX)-loaded micelles.

The zeta potential and hydrodynamic diameter of the mPEG-CS(SH)-CHO micelles play an important role in determining their in vitro and in vivo behaviors [33]. As shown in Figure 4, the mean sizes of the bare and PTX-loaded micelles measured by the DLS method were 110 ± 4.5 nm and 158 ± 6.8 nm, respectively. The increased size of PTX-loaded micelles could be attributed to the encapsulation of the drug. It was reported that particles with sizes in the range of 10 nm to 200 nm could reduce the clearance by the RES to a certain extent and passively target the tumor sites via the EPR effect [5]. Hence, the prepared mPEG-CS(SH)-CHO micelles were suitable for tumor-specific accumulation.

Following the zeta potential determination, the micelles were positively charged, and the values for the blank and drug-loaded micelles were +28.6 ± 0.2 mV and +26.9 ± 0.1 mV, respectively. The difference in value could be attributed to the change of particle size and surface charge density. The relatively high zeta potential implied the high stability of the system in the blood stream. Moreover, the particles with positive charge might have enhanced endocytosis in the cells [34]. Compared with the bare ones, the PTX-loaded micelles were much larger in size and exhibited a lower zeta potential, which could be explained by the encapsulation of the drug.

#### 3.4.2. TEM Observation

**Figure 5:**
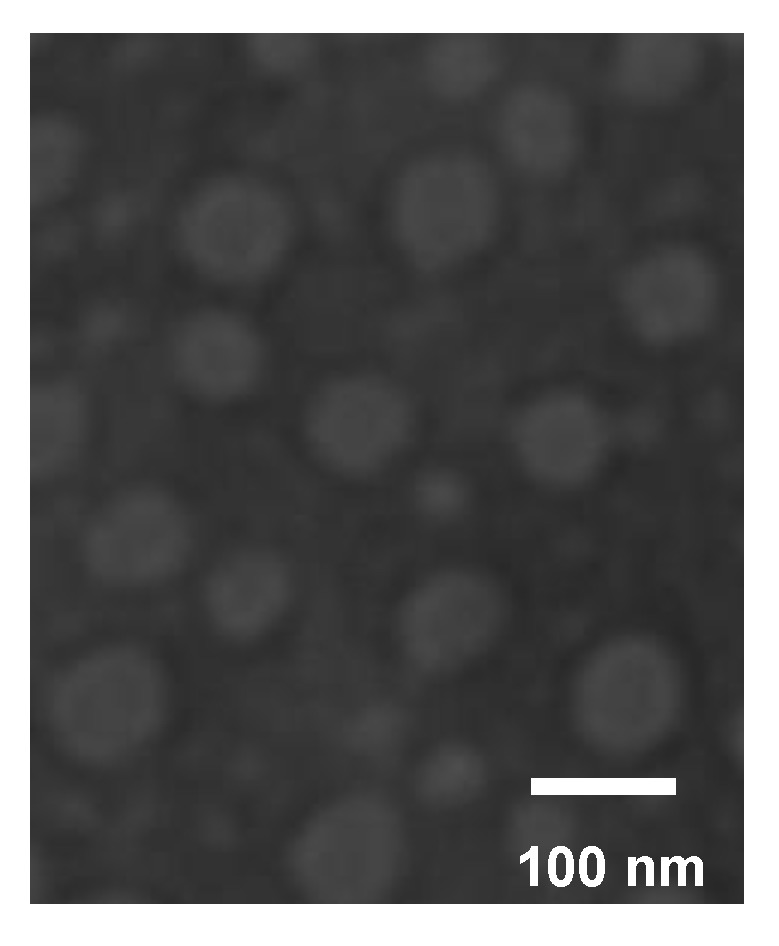
TEM micrograph of PTX-loaded mPEG-CS(SH)-CHO micelles.

The morphology of the PTX-loaded mPEG-CS(SH)-CHO micelles was investigated by TEM observation. As illustrated in Figure 5, the micelles were uniform and displayed a spherical morphology. The particle size estimated from TEM was about 117 nm, which was much smaller than that measured by the DLS method. The possible explanation might be the existence state of the micelles, i.e., the dehydration state and the hydration state, respectively. More exactly, the dehydration process was used during the sample preparation for the TEM experiment, and this might have led to the shrinking of the micelles [35].

#### 3.4.3. XRD Analysis

**Figure 6:**
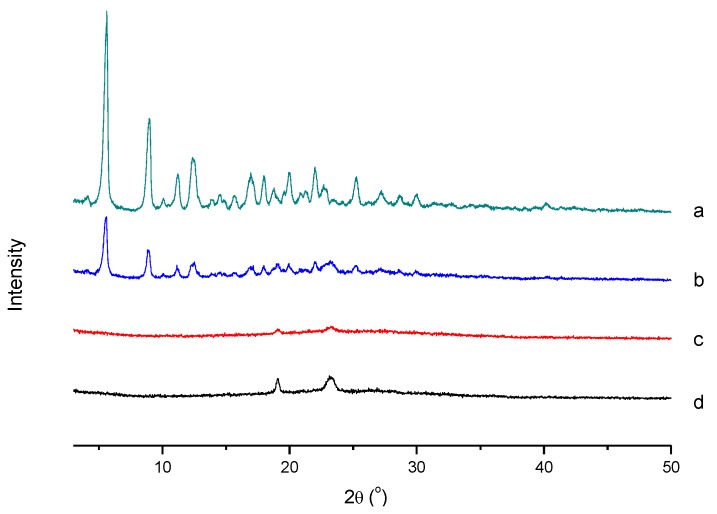
XRD patterns of (**a**) PTX, (**b**) physical mixture of PTX and blank micelles, (**c**) blank micelles, and (**d**) PTX-loaded micelles.

The status of the PTX loaded in the micelles was evaluated by an XRD study, and the results are shown in Figure 6. It was obvious that PTX exhibited characteristic peaks at 2θ of 5.62°, 8.96°, 11.20°, and 12.41°, and there were numerous peaks in the range of 15° to 30°. For the blank micelles, the characteristic peaks of mPEG were observed at 2θ of 19.09° and 23.25°, which also indicated the successful graft of mPEG onto the backbone of CS [36]. The typical peaks of both PTX and blank micelles still existed in the diagram of their physical mixture. While the drug-loaded micelles presented a pattern similar to that of the bare ones, there were no crystal peaks of PTX. It could be deduced that PTX dispersed in the micelles in a non-crystalline state.

### 3.5. Protein Adsorption

Considering the positive charges on the prepared micelles, the protein adsorption of the micelles was estimated using BSA as the model protein. From the results, the BSA adsorption of CS was as high as 31.3%, while CS(SH)-CHO showed a much stronger interaction with BSA, of 59.3%. This could be explained by the fact that the isoelectric point of BSA is 4.7, in PBS 7.4. BSA was negatively charged, and it could be easily adsorbed on to the surface of the positively charged CS. After being modified by CHO-COOH and NAC, the obtained CS(SH)-CHO with enhanced hydrophobicity would have a significantly increased affinity to proteins, which was in agreement with the previous report [37]. However, after mPEGylation, the protein adsorptions of mPEG-CS(SH)-CHO decreased dramatically, to a value as low as 13.8%. This could be attributed to the introduction of mPEG, which has extremely low protein adsorption affinity itself (0.88%). More specifically, mPEG was electrically neutral over a wide pH range and might have formed a hydration shell around the particles. These properties make mPEG block the electrostatic interactions between the micelles and the protein [38]. Moreover, as a long chain polymer, mPEG could provide high elastic repulsion energy to prevent protein adsorption [39]. Since it was believed that the protein adsorption of particles was correlated with their circulation times in the blood [40], it could be deduced that the mPEG-CS(SH)-CHO micelles would extend the circulation time of the drug, and therefore enhance the EPR effect to improve the bioavailability of the drug. 

### 3.6. In Vitro Redox-Responsive Drug Release

**Figure 7:**
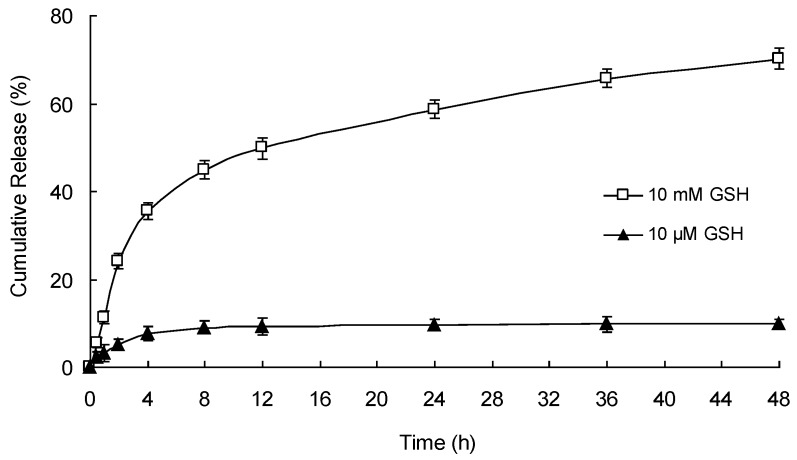
Release of PTX from PTX-loaded mPEG-CS(SH)-CHO micelles under different levels of glutathione (GSH).

The release of PTX from mPEG-CS(SH)-CHO micelles was studied under different levels of GSH (10 μM and 10 mM). The cumulative drug release curves were plotted against time in Figure 7. In the presence of 10 μM of GSH, less than 10% of PTX was released from mPEG-CS(SH)-CHO micelles during 48 h, which indicated the stability of the micelles in the blood stream. However, in the presence of 10 mM of GSH, the released amount of PTX raised significantly, with more than 50% of PTX released within 24 h and up to 70% of PTX released within 48 h. It was inspiring to see that the release rate of PTX was dependent on the concentration of GSH. More exactly, GSH triggered the drug release. With the activation of GSH, the disulfide bonds in the micelles were cleaved rapidly, and the compact micelles disassembled to release PTX. It is reasonable to infer that the PTX-loaded micelles would be considerably stable before reaching tumor sites and could achieve rapid drug release in tumor cells [41]. For the tumor-specific drug release behavior, the micelles might be an ideal redox-responsive drug delivery system.

### 3.7. In Vitro Cytotoxicity

**Figure 8:**
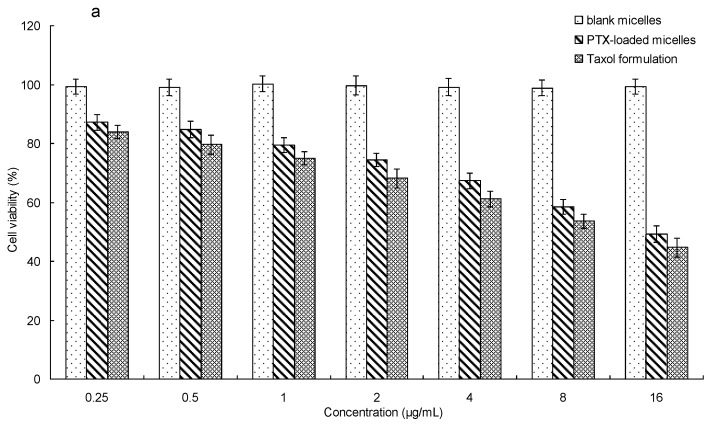

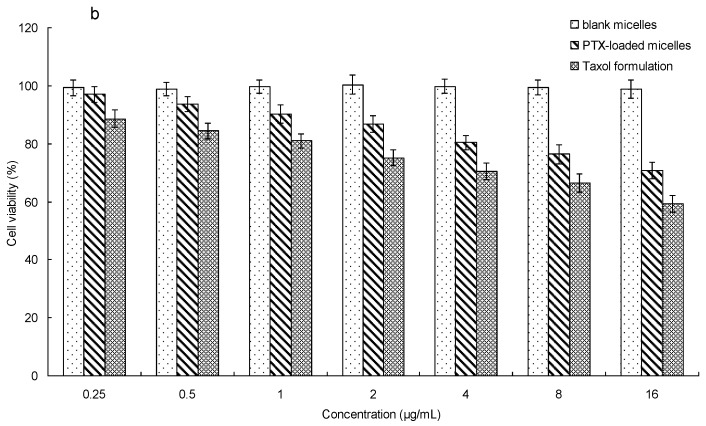
In vitro cytotoxicity of PTX-loaded mPEG-CS(SH)-CHO micelles against (**a**) MCF-7 cells and (**b**) peripheral blood mononuclear cells (PBMC).

An MTT assay was applied to estimate the cytotoxicity of PTX-loaded mPEG-CS(SH)-CHO micelles against MCF-7 cells and PBMC cells. From Figure 8, it was obvious that no less than 98% of the MCF-7 cells and PBMC cells survived after incubation with bare micelles, which indicated the non-toxic nature and excellent biocompatibility of mPEG-CS(SH)-CHO. 

For PBMC cells, the PTX-loaded mPEG-CS(SH)-CHO micelles exhibited notably lower cytotoxicity than the Taxol formulation, which might be explained by the redox-sensitivity of the micelles. After being internalized into the cells, the disulfide bonds in mPEG-CS(SH)-CHO micelles were stable in the cytoplasm of PBMC cells (with low GSH concentration), and this would lead to a small amount of PTX released from the micelles. For MCF-7 cells, both PTX formulations exhibited comparable cytotoxicity at the same concentration of the drug. This might be explained by the rapid intracellular PTX release of the micellar system. More exactly, GSH in the cytoplasm of MCF-7 cells could facilitate the drug release from the PTX-loaded micelles, so as to enhance the antitumor effect of the drug [42]. It was inspiring that the cytotoxicity of PTX-loaded micelles against normal cells (PBMC cells) was much lower than that against cancer cells (MCF-7 cells). These findings suggested that mPEG-CS(SH)-CHO micelles might be a potential delivery system for PTX.

### 3.8. In Vivo Antitumor Efficacy

**Figure 9:**
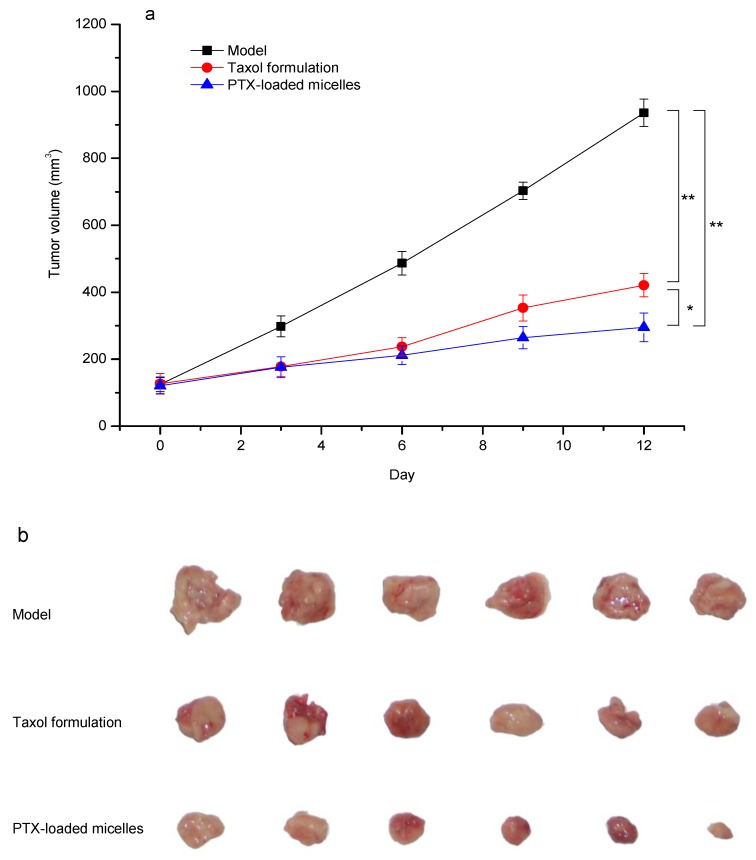
In vivo antitumor activity of PTX-loaded mPEG-CS(SH)-CHO micelles: (**a**) tumor volume growth curves after various treatments (* *p* < 0.05; ** *p* < 0.01); (**b**) photos of tumors excised from different groups.

The anticancer potential of PTX-loaded mPEG-CS(SH)-CHO micelles was further corroborated by performing a tumor regression study. As illustrated in Figure 9, tumors in the model group grew rapidly. In comparison, the groups treated with PTX formulations exhibited a remarkable reduction in tumor size. Moreover, as expected, the PTX-loaded micelles exhibited a superior tumor inhibitory effect than Taxol, with TIR of 67.3% vs. 53.8%. The noticeable tumor suppression effect of PTX-loaded micelles could be explained by the superior properties of the mPEG-CS(SH)-CHO micellar system. More specifically, the mean size in the range of 100–200 nm could grant the micelles a passive targeting effect. The modification by mPEG could shield the micelles from rapid phagocytosis by RES and prolong the circulation time to enhance the EPR effect. Moreover, the positive charge could facilitate the attachment of micelles to the tumor surface (negatively charged) via the electrostatic interaction [43]. And it was much easier for positively charged particles to penetrate into the tumor cells [44]. On the other hand, after entering the tumor, the disulfide bonds-employing micelles could disassemble rapidly and release the drug effectively under a high level of GSH [45,46]. In contrast, they were stable at normal physiologic conditions, and this would reduce the toxicity to the body. In summary, the tumor-specific accumulation and drug release could lead to a superior tumor inhibition effect. The PTX-loaded mPEG-CS(SH)-CHO micelles might be a promising carrier system for delivery of PTX.

## 4. Conclusions

In this study, a redox-responsive copolymer of mPEG-CS(SH)-CHO has been successfully designed and synthesized by linking mPEG, CHO, and NAC to the backbone of CS. Due to its amphiphilic structure, mPEG-CS(SH)-CHO could spontaneously form micelles and solubilize PTX within the hydrophobic core. The optimized PTX-loaded micelles were successfully prepared with a mean size of 158 nm and a zeta potential of +26.9 mV. An in vitro drug release study revealed the redox-sensitivity of the micelles and showed that they could rapidly release the drug in the presence of high levels of GSH. Moreover, the PTX-loaded micelles possessed excellent anticancer effect in vivo. Based on the above results, the initial hypothesis has been confirmed. It can be concluded that the mPEG-CS(SH)-CHO is a promising redox-responsive drug delivery carrier for the controlled release of PTX.
